# Acute Inner Ear Complications of Stapes Surgery: Value of Delayed Postcontrast 3D‐FLAIR MRI Sequences

**DOI:** 10.1002/oto2.70136

**Published:** 2025-06-03

**Authors:** Jean Fanet, Sylvain Bourdoncle, Guillaume Poillon, Mary Daval, Daniel Levy, Denis Ayache, Stéphane Gargula

**Affiliations:** ^1^ Department of Otolaryngology Hôpital Fondation Adolphe de Rothschild Paris France; ^2^ Department of Neuroradiology Hôpital Fondation Adolphe de Rothschild Paris France; ^3^ Department of Neuroradiology Hôpital Pierre‐Paul‐Riquet, CHU Toulouse Toulouse France; ^4^ ENT‐HNS Department APHM, CNRS, IUSTI, La Conception University Hospital, Aix Marseille Univ Marseille France

**Keywords:** inner ear, magnetic resonance imaging, otosclerosis, stapes surgery

## Abstract

**Objective:**

To describe and assess the usefulness of delayed postcontrast three‐dimensional fluid‐attenuated inversion recovery (3D‐FLAIR) sequences on 3‐Tesla (3 T) magnetic resonance imaging (MRI) in patients presenting with acute inner ear complications after stapes surgery.

**Study Design:**

Case series.

**Setting:**

French tertiary referral center.

**Methods:**

The clinical records and imaging of patients who underwent delayed postcontrast 3D‐FLAIR MRI sequences for labyrinthine complications after stapes surgery, performed between January 2019 and April 2023, were retrospectively reviewed.

**Results:**

A total of 712 patients underwent stapes surgery between January 2019 and December 2023. Eight patients (1.12%) were included in the study, with a median age of 52 years (interquartile range 40‐54). After the surgery, seven patients presented with vertigo and sensorineural hearing loss (SNHL), and one patient presented with only vertigo with complete areflexia on caloric testing. Computed tomography (CT) of the temporal bone showed a slightly excessive penetration of the prosthesis (>1 mm) into the vestibule in one patient and a periprosthetic granuloma in another patient. CT was normal for six patients. Delayed postcontrast 3D‐FLAIR MRI sequences showed blood‐labyrinth barrier (BLB) impairment in the cochlea, the vestibule, and the semicircular canals in seven patients. No endolymphatic hydrops were found, but one patient presented with utricular collapse, and the saccule was not visible in three other patients.

**Conclusion:**

Delayed postcontrast MRI sequences may reveal BLB impairment and help analyzing the endolymphatic compartment in cases of SNHL or vestibular disorders after stapes surgery. Those sequences could help uncovering the causes of such events.

Sensorineural hearing loss (SNHL) and persistent vertigo are the main concerns after stapes surgery.^1^, ^2^ Imaging plays an important role in evaluating acute complications associated with stapes surgery. Computed tomography (CT) can reveal an overpenetrating prosthesis (>1 mm into the vestibule), pneumolabyrinth, or the presence of a reparative granuloma.^3^ When CT findings are inconclusive, gadolinium‐enhanced magnetic resonance imaging (MRI) may be helpful in suggesting reparative granuloma extending to the vestibule, labyrinthitis, or intralabyrinthine hemorrhage.^4^ Developed in the 2000s, delayed postcontrast three‐dimensional fluid‐attenuated inversion recovery (3D‐FLAIR) imaging sequences in MRI, performed 4 hours after intravenous injection of gadolinium, allow a fine analysis of the perilymphatic and endolymphatic compartments.^5^, ^6^ Some studies have shown that otosclerosis patients, outside of a surgical context, may have increased perilymph enhancement on delayed postcontrast 3D‐FLAIR sequences, suggesting a focal permeability of the blood‐labyrinth barrier (BLB) in otosclerotic lesions.^7^ Some authors reported that the prevalence of hydrops is greater in patients with otosclerosis, but it is debated.^8^, ^9^, ^10^ The aim of this study was to describe images obtained with delayed postcontrast 3D‐FLAIR sequences in patients with acute postoperative inner ear complications after stapes surgery, and to discuss the potential value of these sequences in the diagnostic management of these complications.

## Material and Methods

### Patients

The clinical records of consecutive patients who underwent delayed postcontrast MRI for acute inner ear complications after stapes surgery performed between January 2019 and December 2023 at a French tertiary referral center were retrospectively reviewed.

The primary surgery was performed according to the same procedure, regardless of the surgeon. Under general anesthesia and using a minimal endaural approach, the tympano‐meatal flap was raised to the annulus. After opening the tympanic cavity, Rosen's notch was curetted to allow good exposure of the stapes pyramid. The incudostapedial joint was separated, and the tendon and posterior branch of the stapes were sectioned using a diode laser. The superstructure of the stapes was removed. Stapedotomy was performed using a laser or a manual perforator if the facial nerve was dehiscent. 4.5 × 0.6 mm Teflon prostheses (Causse) are the standard prostheses used in our center for stapes surgery. 4.5 × 0.5 mm titanium prostheses (BigEasy, Medtronic) were used in selected cases (narrow anatomy, subjective need for tighter crimping of the loop).

After primary surgery, patients who presented with vertigo and/or hearing loss with or without tinnitus underwent a full clinical examination with videonystagmoscopy and audiometric testing with pure‐tone average (PTA) hearing levels measured by bone conduction (BC). Depending on the clinical presentation, availability, and the clinician's assessment, a video head impulse test (vHIT) and/or videonystagmography (VNG) with caloric test may be performed. Inner ear complications were defined as SNHL with a bone‐conduction threshold reduction of >20 dB with or without vertigo, or isolated persistent vertigo with either areflexia on caloric testing or canal dysfunction on vHIT. Patients with acute inner ear complications underwent CT of the temporal bone and delayed postcontrast MRI focused on the inner ear. These patients were included in the study.

For each patient, the following data were collected: sex, age, side involved, history of stapes surgery on the contralateral ear, PTA hearing levels of air conduction (AC) and BC before surgery, preoperative symptoms (vertigo, tinnitus), BC hearing level on postoperative audiogram, results of any complementary examinations (VNG, vHIT), and evolution of symptoms and audiometry during follow‐up.

AC and BC averages (PTA) were calculated as the mean hearing thresholds at 0.5, 1, 2, and 3 kHz (or 4 kHz if 3 kHz was not available), following the guidelines of the American Academy of Otolaryngology–Head and Neck Surgery (AAO‐HNS).^11^ Air bone gap was calculated as BC PTA minus AC PTA.

### Imaging Protocol and Analysis

MRI examinations were performed at 3 T (3T Elition® Philips Healthcare, 3T Galan® Canon Medical) with a 32‐ or 64‐channel head coil. The imaging protocol focused on the inner ear included at least high‐resolution 3D T2‐WI and thin slices 3D‐FLAIR sequences, both performed 4 hours after a single intravenous dose of gadobutrol (Gadovist®, Bayer Healthcare, at a dose of 0.1 mL per kg). Data on the delay between surgery and MRI were collected. All patients included in the study underwent a noninjected high‐resolution CT of the temporal bone. Preoperative examinations, if available (CT and/or MRI), were also analyzed.

For each patient, CT and MRI images were evaluated independently by two neuroradiologists experienced in inner ear imaging blinded to the clinical data.

The primary endpoints were, on MRI, the impairment of the BLB and its systematization (basal turn of the cochlea, cochlea, vestibule, and semicircular canals) and the presence of endolymphatic abnormality (saccular hydrops, cochlear hydrops, utricular hydrops, ampullar hydrops, no visible saccule, and utricular collapse). Those were qualitatively assessed on delayed postcontrast 3D‐FLAIR sequences. On 3D T2‐weighted sequences, the visibility and penetration of the piston, homogeneity of the labyrinthine fluid signal, and presence of any labyrinthine malformation were described.

The site and extent of the otosclerosis (fissula ante fenestram, pericochlear, perivestibular, round window with or without obliteration, and internal acoustic meatus) were described on the CT scan. Associated anomalies such as labyrinthine malformation, calcification of the vestibular aqueduct, dehiscence of the semicircular canals, and ossicular anomaly were also collected. Postoperatively, the penetration depth of the prosthesis into the vestibule (more or less than 1 mm), periprosthetic granuloma, or pneumolabyrinth was reported if present.

Patients provided consent for the use of their data. This study was approved by the institutional review board “IRB 00012801” under the validation number ID “CE_20220726_3_SGA.” The STROBE guidelines were used for reporting.^12^

## Results

### Patients

Between January 2019 and December 2023, 712 patients underwent stapedotomy or stapedectomy at our center. Eight patients (1.12%) presented with acute inner ear complications after the stapes surgery. The clinical data for each patient are listed in Table 1.

**Table 1:** Patient's Clinical Characteristics

Case	Sex	Age, y	Side of otosclerosis (operated ear)	History of stapes surgery on the other ear	Postoperative vertigo	Changes in BC‐HL level of the operated ear (preoperative BC‐HL/postoperative BC‐HL)	Postoperative vHIT	Postoperative caloric test, %
1	F	54	R (R)	N	Y	−23 (15/38)	NA	NA
2	F	50	R (R)	N	Y	−25 (15/40)	Dysfunction of all semicircular canals	Complete areflexia (100%)
3	M	40	B (L)	N	Y	−40 (30/75)	NA	NA
4	F	62	B (L)	Y	Y	0 (35/35)	Lateral semicircular canal dysfunction	Complete areflexia (100%)
5	M	40	L (L)	N	Y	−75 (15/90)	Dysfunction of all semicircular canals	NA
6	F	56	B (R)	Y	Y	−35 (35/70)	NA	NA
7	F	47	B (R)	N	Y	−30 (20/50)	Normal	Partial areflexia (60%)
8	M	54	B (L)	N	Y	−55 (35/90)	Dysfunction of all semicircular canals	NA

Abbreviations: B, bilateral; BC‐HL, bone‐conduction‐hearing level; F, female; L, left; M, male; N, no; NA, not applicable; R, right; vHIT, video head impulse test; y, year; Y, yes.

Five patients were women, and three were men. The median age of the patients was 52 years (interquartile range [IQR] 40‐54; min 40‐max 62). Five patients had bilateral otosclerosis. Two of these patients had a history of stapes surgery on the other ear. A Teflon prosthesis was used for all patients. All the patients presented with vestibular symptoms and contralateral nystagmus. Seven patients had a worsening of BC hearing levels of at least 20 dB. One patient presented only with vestibular symptoms. VNG with caloric testing was performed for three patients: two had complete areflexia, and one had 60% hyporeflexia. The vHIT was performed in five patients: one was normal, three had dysfunction of all semicircular canals, and one had an isolated dysfunction of the lateral semicircular canal.

Two patients underwent revision surgery combined with intravenous antibiotics and corticosteroid therapy (patients 3 and 5). Three patients were treated with intravenous antibiotics and corticosteroids (patients 1, 4, and 6). Two patients received intravenous antibiotics, intravenous corticosteroids, and intratympanic corticosteroids (patients 2 and 8). One patient received the same treatment and hyperosmolar intravenous therapy (patient 7).

Two patients had completely recovered from hearing loss and vertigo 2 months after surgery. Four patients recovered well from vertigo but not from hearing loss. One patient did not recover from vertigo or hearing loss 2 months after surgery. One patient was lost to follow‐up.

### MRI Analysis

**Figure 1:**
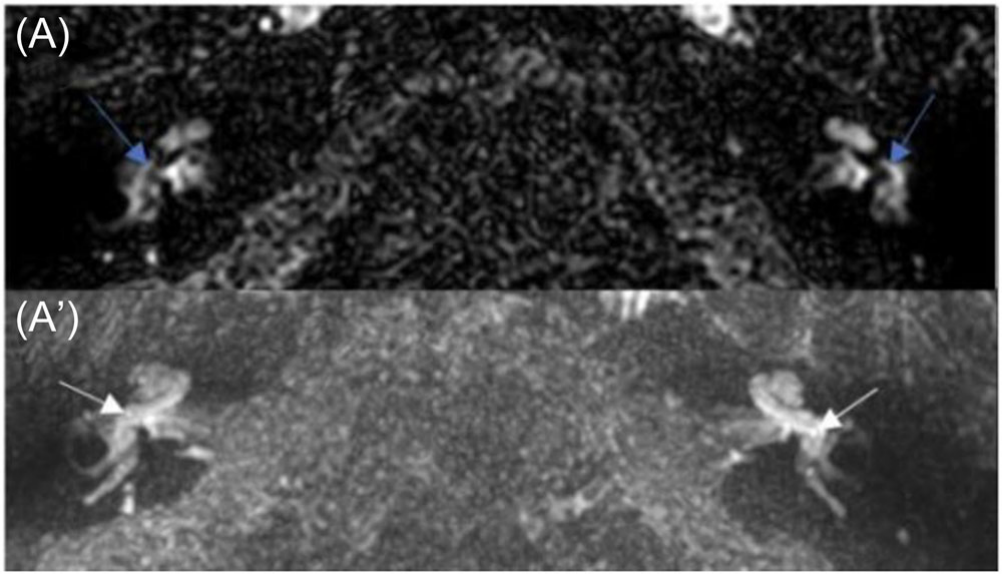
Case 3, preoperative magnetic resonance imaging. (A) The endolymphatic compartment is clearly visible and appears normal (blue arrow showing saccule). (A′) Slight blood‐labyrinth barrier impairment of the basal turn of the cochlea (white arrow).

After surgery, all patients underwent MRI examinations including a postcontrast 4 hours delayed 3D‐FLAIR sequence dedicated to labyrinth examination. The median time between surgery and MRI was 24.5 days [IQR 13‐37; min 3‐max 79]. For patients who underwent revision surgery, the MRI was performed after the initial surgery but before the revision. One patient also underwent delayed postcontrast MRI examination before surgery (Figure 1).

**Figure 2:**
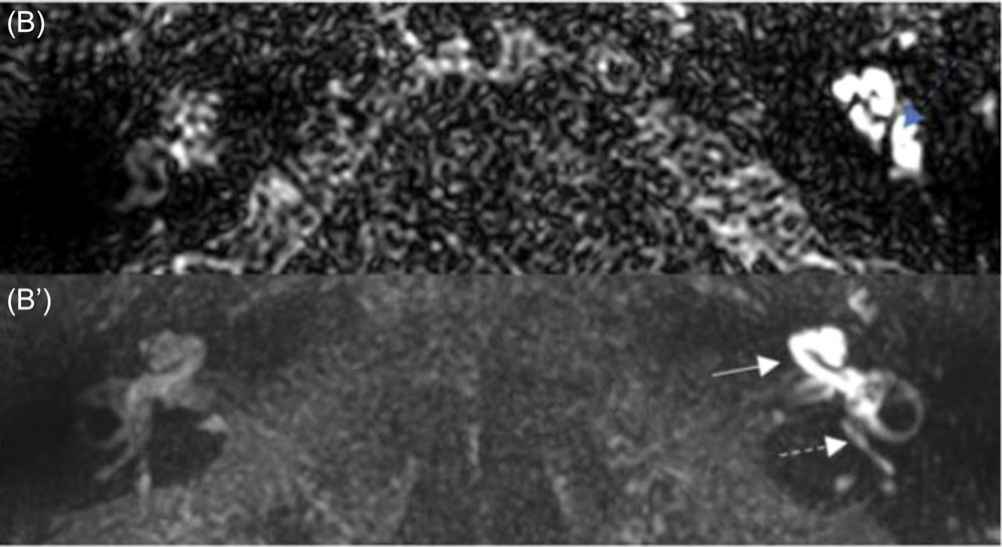
Case 3, postoperative magnetic resonance imaging. (B) No visible saccule on the left‐operated ear (blue dashed arrow). (B′) Intense blood‐labyrinth barrier impairment of the cochlea (white arrow), the vestibule, and semicircular canals (white dashed arrow).

**Figure 3:**
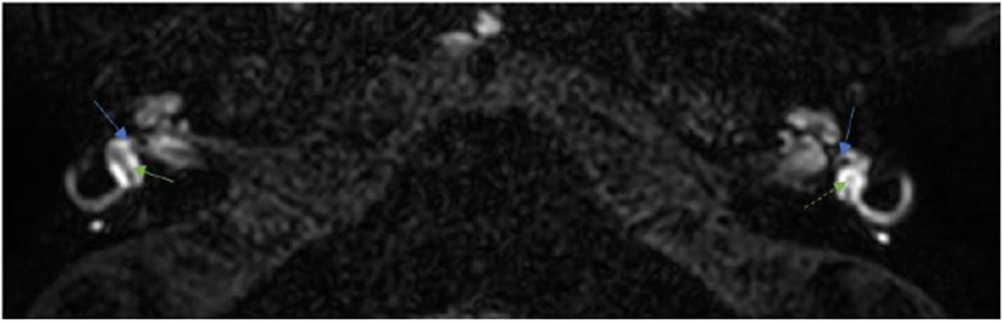
Case 4. Left utricular collapse (green dashed arrow). Right utricle (green arrow). Visible saccules (blue arrows). Bilateral BLB impairment of the basal turn of the cochlea is also noticeable.

**Figure 4:**
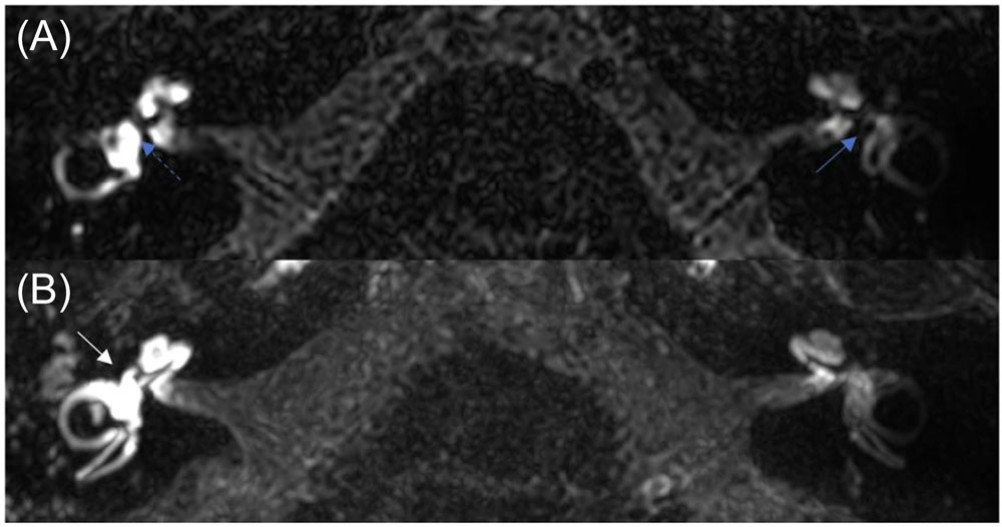
Case 1. (A) No visible saccule on the right operated ear (blue dashed arrow). Left saccule (blue arrow). (B) Intense BLB impairment of the cochlea, the vestibule and the semicircular canals (white arrow).

Seven patients presented with an intense BLB impairment of the cochlea, vestibule, and semicircular canals (Figures 2B′ and 3B). Patient 4, who had no hearing loss, was the only patient without widespread BLB impairment. Among the five patients with bilateral disease on CT scans, BLB impairment of the basal turn of the contralateral cochlea was found in three of them, and notably on the preoperative MRI of patient 3 (Figures 1A′ and 4).

Regarding the endolymphatic compartment, no hydrops were encountered in any of the eight patients. One patient had utricular collapse (Figure 4), and three patients had no visible saccule (2, 3 and 2, 3).

In patient 5, where the prosthesis penetrated the vestibule by more than 1 mm, MRI enabled us to ensure that the prosthesis did not injure the saccule.

The MRI findings, interventions, and recovery of each patient are displayed in Table 2.

**Table 2:** Details on Complication, Magnetic Resonance Imaging (MRI) Findings, Intervention, and Recovery

Case	Complication *changes in BC‐HL level of the operated ear	MRI findings	Intervention	Recovery
1	Vertigo	Widespread BLB impairment	Intravenous antibiotics and corticosteroids	Good recovery from hearing loss and vertigo (at 2 mo)
	SNHL *−23 dB	No visible saccule		
2	Vertigo	Widespread BLB impairment	Intravenous antibiotics, intravenous corticosteroids, and intratympanic corticosteroids	No recovery of vertigo or hearing loss (>1 y)
	SNHL *−25 dB			
3	Vertigo	Widespread BLB impairment	Revision surgery (periprosthetic reparative granuloma) combined with intravenous antibiotics and corticosteroid therapy	No recovery of hearing loss (>1 y) Good recovery from vertigo (at 1 mo)
	SNHL *−40 dB	No visible saccule		
4	Vertigo	Utricular collapse, no BLB impairment	Intravenous antibiotics and corticosteroids	NA
5	Vertigo	Widespread BLB impairment	Revision surgery (overpenetrating piston in the vestibule) combined with intravenous antibiotics and corticosteroid therapy	No recovery of hearing loss (>1 y) Good recovery from vertigo (at 2 mo)
	SNHL *−75 dB			
6	Vertigo	Widespread BLB impairment	Intravenous antibiotics and corticosteroids	No recovery of hearing loss (>1 y) Good recovery from vertigo (at 2 mo)
	SNHL *−35 dB			
7	Vertigo	Widespread BLB impairment	Intravenous antibiotics, intravenous corticosteroids, and hyperosmolar intravenous therapy	Good recovery from hearing loss and vertigo (at 1 mo)
	SNHL *−30 dB			
8	Vertigo	Widespread BLB impairment	Intravenous antibiotics, intravenous corticosteroids, and intratympanic corticosteroids	No recovery of hearing loss (1 y) Good recovery from vertigo (at 15 d)
	SNHL *−55 dB	No visible saccule		

Abbreviations: B, bilateral; BC‐HL, bone‐conduction‐hearing level; BLB, blood‐labyrinth barrier; F, female; L, left; M, male; N, no; NA, not applicable; R, right; SNHL, sensorineural hearing loss; Y, yes.

### CT Scan Analysis

All patients had hypodensity on preoperative CT scans, at least at the fissula ante fenestram. One patient also presented with round window otosclerosis involvement (without obliteration), and one patient had internal acoustic meatus and pericochlear involvement. No other anomalies were observed on preoperative scans.

Postoperatively, of the eight CT scans analyzed, one showed a piston that was slightly too penetrating into the vestibule (1.1 mm), and one suggested a periprosthetic granuloma. No other anomaly was observed.

## Discussion

Our study illustrates the usefulness and contribution of delayed postcontrast 3D‐FLAIR in the management of patients with neurosensory complications after stapes surgery. This imaging tool enabled us to visualize inner ear damage in all our patients. Most patients suffered damage to the BLB, suggested by excessive perilymph enhancement, and half of the patients presented images suggesting impairment of the endolymphatic compartment.

Surgical techniques for otosclerosis have evolved over the last few decades, with stapedectomy gradually giving way to stapedotomy. In addition to the beneficial results in terms of hearing, stapedotomy has reduced the incidence of SNHL postoperatively.^13^ Although infrequent, postoperative SNHL is a dreaded complication. In our center, between January 2019 and December 2023, 1.12% of patients who underwent stapedotomy or stapedectomy presented with acute postoperative inner ear complications, and four of them (0.56%) had definitive SNHL. This result is consistent with data in the literature. Depending on the study, significant SNHL can be encountered in 0.5% to 5.9% of patients.^1^, ^14^, ^15^ Immediate postoperative vertigo occurs in a highly variable and sometimes significant number of cases (ranging from 3.4% to 70%).^16^ Vestibular symptoms rarely persist for several months after surgery.^17^ In our cohort, six patients followed up for more than a year were free of vestibular symptoms after 2 months.

Imaging may help identify the cause of these complications. The first examination carried out is usually a CT scan of the temporal bone. It can reveal an overpenetrating prosthesis into the vestibule, a periprosthetic granuloma, or a persistent pneumolabyrinth secondary to a perilymphatic fistula.^18^ But CT is often unable to identify any of these specific causes and does not provide the notion of potential injury to the membranous labyrinth due to an overpenetrating prosthesis or intraoperative manipulation.^19^, ^20^ In our cohort, a possible cause was suggested on CT in only two of eight patients. One patient had an opacity encompassing the prosthesis, suggestive of reparative granuloma. The other patient had a slightly overpenetrating prosthesis into the vestibule (1.1 mm). However, postcontrast 3D‐FLAIR sequences showed that the saccule was not damaged by this overpenetrating prosthesis. In both cases, the patients underwent revision surgery. They recovered well in terms of vertigo, but not in terms of hearing.

MRI may be a good complementary examination in patients for whom CT proves to be noncontributory. It could reveal other complications, such as intralabyrinthine hemorrhage, intravestibular reparative granuloma, or labyrinthitis.^4^, ^18^ Because conventional inner ear MRI lacks precision for labyrinth exploration, new MRI protocols have emerged over the past decade, based on the completion of a 4 4‐hour‐delayed intravenous gadolinium‐enhanced 3D‐FLAIR sequence, allowing a detailed analysis of both endolymphatic and perilymphatic compartments on 3 T MRI.^21^ That is particularly relevant for detecting endolymphatic hydrops (EH) in Ménières' disease as well as for identifying other labyrinthine anomalies such as vestibular atelectasis or BLB impairments when investigating various acute or recurrent cochlear and/or vestibular symptoms.^5^, ^22^, ^23^, ^24^, ^25^

In 7/8 patients in our study, we identified a BLB impairment of the cochlea, the vestibule, and the semicircular canals. The only patient without diffuse BLB impairment had no SNLH and had an MRI performed 79 days after surgery, which may have influenced the results.

Rangheard et al, using precontrast and postcontrast T1‐WI sequences combined with T2‐WI, described a high enhancement of labyrinth fluids only in 1/11 patients who presented with SNHL after stapedectomy.^4^ Delayed postcontrast 3D‐FLAIR sequences therefore appear to be more sensitive to identify BLB impairment.^26^ In our series, we have also noted, in the contralateral ears of patients 7 and 8 with otosclerosis and no history of stapes surgery, a BLB impairment of the basal turn of the cochlea. The same findings were made in both ears of patient 4 (history of surgery on the right side and acute vestibular impairment without hearing loss at the time of surgery). Naganawa et al demonstrated this increase in signal intensity ratio in the basal turn of the cochlea in patients with otosclerosis, using delayed postcontrast 3D‐FLAIR sequences.^7^ According to Lombardo et al, this increase in signal intensity in otosclerosis, coinciding with an increase in BLB permeability, could be secondary to a phenomenon of hyperemia and venous stasis in the spongiotic endochondral layer.^26^ Laine et al also demonstrated this BLB impairment in otosclerosis patients with no history of stapes surgery and showed that these MRI‐visible abnormalities were not correlated with vestibular symptoms or SNHL.^10^

Some authors suggest that patients with otosclerosis have an increased risk of EH, whether or not they have undergone stapes surgery.^8^, ^27^ Endolymphatic compartment impairment may be related, outside a surgical context, to obstruction of the vestibular aqueduct by an otosclerotic focus and, in a context of stapes surgery, to direct trauma to the membranous labyrinth.^28^, ^29^ Regarding the endolymphatic compartment, we found no evidence of vestibular or cochlear hydrops in our patients. Patient 4, who presented with vertigo and nystagmus for several weeks with complete areflexia on caloric test but no SNHL, underwent MRI more than 2 months after surgery. This delayed postcontrast MRI showed, apart from a nonspecific impaired BLB of the basal turn of the cochlea, a utricular collapse on the side of the operated ear. Eliezer et al and Attyé et al identified vestibular atelectasis on delayed postcontrast MRI as a possible cause of vestibular dysfunction.^22^ They also observed the absence of the saccule in a subgroup of patients with Ménières' disease.^22^, ^30^ By studying clinical and radiological characteristics of patients with no visible saccule, they found that this anomaly seems to be multifactorial and suggest two main mechanisms: saccular collapse or saccular fistula. They report the case of a patient with otosclerosis who presented with postoperative SNHL with no visible saccule on delayed postcontrast 3D‐FLAIR sequences. They suggest that, in this context of stapes surgery, saccular fistula is the most likely mechanism by trauma of the saccule by the prosthesis.^31^ Although there are no data in the literature concerning the interpretation of 3D‐FLAIR sequences in patients with titanium prostheses, they could give rise to an artifact that could hamper interpretation. It would therefore be interesting to study the 3D‐FLAIR sequences of patients with titanium prostheses.

Our study has several limitations. First, as a retrospective analysis, it may be subject to bias in estimating the proportion of inner ear complications. Some patients may have been lost to follow‐up or may not have undergone an MRI. The aim of this study was descriptive, and the small number of patients did not allow us to correlate clinical data with imaging data, nor perform comparative statistics. However, the rarity of inner ear complications makes this limitation difficult to avoid. It is important to note that delayed postcontrast 3D‐FLAIR MRI is not routinely performed for stapes surgery patients without complications. Therefore, in this retrospective study, we lack comparative imaging data from routine stapes operations, which limits our ability to definitively label BLB impairment as a prognostic finding. It would be interesting to perform delayed postcontrast MRIs carried out in patients who had recently undergone stapes surgery but had no postoperative events, to ascertain the causality between damage to the BLB and symptomatology. Also, preoperative MRI, which was only available in 1/8 patient, could provide a comparator and an indication of the state of the endolymphatic compartment before surgery. The timing of MRI scans in relation to treatments varied among patients, which could potentially influence the imaging findings. This variability could be a limitation of our study and should be considered when interpreting the results.

This study suggests that inner ear MRI with delayed postcontrast 3D‐FLAIR is highly sensitive in cases of acute inner ear complications after stapes surgery and makes it possible to describe excessive enhancement of the perilymphatic compartment and structural anomalies of the endolymphatic compartment.

Although our findings suggest that delayed postcontrast 3D‐FLAIR MRI can visualize inner ear damage, its clinical utility in guiding management decisions, such as revision surgery, remains to be determined. Given their nonspecific nature, these findings must be correlated with the clinical presentation. It is conceivable, for example, that the absence of visibility of the saccule is linked to trauma to the saccule. The two patients who underwent revision surgery had a stapedovestibular granuloma and a prosthesis suspected of being too long. These two complications were noticeable on the CT scan, although the absence of saccular lesions in the patient with the excessively long prosthesis was an interesting finding from the MRI. This analysis will first have to be carried out on a case‐by‐case basis, given the rarity of this complication. These images have never been described in the literature, and before any therapeutic conclusions can be drawn, it will be necessary to identify patterns using these images and further data on the subject. The application of these images and their ability to modify patient care remains to be demonstrated. Future studies must provide the findings in delayed postcontrast MRI in patients who have undergone stapes surgery, but without inner ear complications.

## Conclusion

Delayed 3D‐FLAIR postcontrast MRI sequences enabled us to visualize abnormalities of the inner ear in all our patients, whereas the CT scan showed an abnormality in only two patients. The high sensitivity of these sequences in identifying BLB impairment and their utility in analyzing the endolymphatic compartment highlight their potential role in uncovering the causes of SNHL or vestibular disorders after stapes surgery. Future research with appropriate controls is needed to determine if these imaging findings can guide clinical decision‐making or alter patient management.

Stéphane Gargula and Jean Fanet had full access to all the data in the study and took responsibility for the integrity of the data and the accuracy of the data analysis.

## Author Contributions

**Jean Fanet**, conceived and designed the analysis; collected the data; contributed data or analysis tools; performed the analysis; wrote the paper. **Sylvain Bourdoncle**, conceived and designed the analysis; collected the data; contributed data or analysis tools; performed the analysis; wrote the paper. **Guillaume Poillon**, conceived and designed the analysis; collected the data; contributed data or analysis tools; performed the analysis; reviewed the paper. **Mary Daval**, contributed data or analysis tools. **Daniel Levy**, contributed data or analysis tools. **Denis Ayache**, conceived and designed the analysis; contributed data or analysis tools; review the paper. **Stéphane Gargula**, conceived and designed the analysis; collected the data; contributed data or analysis tools; performed the analysis; reviewed the paper.

## Disclosures

### Competing interests

The authors declare that there is no conflict of interest.

### Funding source

This research did not receive any specific grant from funding agencies in the public, commercial, or not‐for‐profit sectors.
